# Plastic Venture Builder (PVB): An empirically derived assessment tool to support plastic waste management ventures in low- and middle-income countries

**DOI:** 10.1177/0734242X231180648

**Published:** 2023-06-30

**Authors:** J.B. Grassin, H. Dijkstra

**Affiliations:** 1 ENVR Department, The Hong Kong University of Science and Technology, Hong Kong; 2 Plastic Odyssey Expedition, Nomad Plastic Limited, Hong Kong; 3 Institute for Environmental Studies, Vrije Universiteit Amsterdam, Amsterdam, Netherlands

**Keywords:** Plastic waste management, sustainable entrepreneurship, assessment framework, LMIC

## Abstract

Plastic waste management is a complicated challenge that in recent years has gained attention as a global policy priority. In low- and middle-income countries (LMIC), waste management is heterogeneous and context-specific and many organizations provide needed waste management services, including entrepreneurs. Sustainable entrepreneurs are uniquely positioned to provide these services; however, they face challenges such as limited support system and lack of capacity. The goal of this paper is to understand critical characteristics of successful plastic waste management ventures in LMIC and operationalize those insights into a strategic tool. A wide variety of successful ventures from diverse LMIC contexts are systematically analyzed to identify which factors contribute to their business viability and ability to deliver services. The identified success factors were built into a tool, the Plastic Venture Builder (PVB), based on the multi-criteria analysis methodology. This is validated using empirical cases, tested on projects currently in development and discussed with experts in the field. The results show that political, economic, financial, technological, operational, social, team and legal factors contribute to success; however pathways to success are diverse. We identify a strong team as the most critical factor, whereas financial, political and social factors have the least impact. The PVB can be used by entrepreneurs who want to set up or improve plastic waste management ventures by identifying weak spots or avenues for improvement. The assessment framework can also be used by policy makers, development agencies and financing organizations who want to support or assess waste management initiatives by prioritizing their resources to match the identified critical factors.

## Introduction and background

Adequate waste management is a global priority, as reflected in both Sustainable Development Goal (SDG) 11, Sustainable cities and communities, and SDG 12, Responsible consumption and production, that is not yet achieved on a global scale. Though technologies and processes for managing waste exist, these often are ‘outdated, underutilized or abandoned’ in lower income countries (Willis et al., 2021). The lack of proper waste management systems in these regions has implications for the economy, environment and societal well-being (Bufoni et al., 2016). Thus, improvements to waste management are needed, including both top-down strategies such as international investments and capacity building, and bottom-up projects led by communities, businesses or individuals (Davis and Garb, 2015; Vital Ocean et al., 2020). In the last decade, there has been an influx of attention to the problem of plastic waste specifically such as a call for a United Nations level plastics treaty (Diana et al., 2022). Regulatory tools are critical as they provide guidance, support and investments; however governance strategies are implemented gradually and do not immediately remediate inadequate waste management. In the meantime, services are being provided by bottom-up actors and strengthening and scaling these initiatives can help reach global waste management goals (Chukwuone et al., 2022).

The amount of waste produced in the world continues to grow alongside a concern for the problem of plastic pollution (Kaza et al., 2018; OECD, 2022). On the one hand, this represents a global challenge, on the other, an opportunity. Due to increased public and political attention to plastic, there has been a growth in demand for recycled products, recyclate and waste management services (Barford and Ahmad, 2021). This demand has been noticed and exploited by grassroots organizations, entrepreneurs and small and medium enterprises (SMEs). These ventures often exist outside of or complementary to solid waste management (SWM) offered by governments. Informal waste management is not a new phenomenon and is prevalent in many low- and middle-income countries (LMICs) as a key facet of waste management (Nandy et al., 2015; Véron et al., 2018). However, due to macro trends such as the globalization of the plastic waste trade, there are increasingly more opportunities for individuals and companies to work in the waste sector and tap into value chains (Liu et al., 2018). If new ventures entering the market can deliver needed services such as waste processing while creating a financially viable business model, they can be a significant asset in reaching global waste management goals (Horvath et al., 2018). The aim of this paper is to understand the characteristics of successful plastic waste management ventures in LMICs and build a tool based on these insights to support entrepreneurs in the field.

To date, much research on improving waste management has focused on supporting decision makers in designing effective SWM systems (Allesch and Brunner, 2014; Guerrero et al., 2013; Mwanza and Mbohwa, 2017; Perteghella et al., 2020). In LMICs, SWM is often not a formal service but instead comprises a chain of connected actors including waste pickers, aggregators, recyclers and buyers (Bening et al., 2021; Silva de Souza Lima Cano et al., 2022). Therefore, many traditional SWM decision-support tools that focus on physical infrastructure or technologies miss the complicated human, cultural and political aspects of SWM in LMICs (Gutberlet et al., 2017; Zurbrügg et al., 2014). Waste generation and management are based on ‘culturally specific popular practices’ and technical solutions that may work in one LMIC’s context but cannot be simply duplicated in a different region (Dumbili and Henderson, 2020: 6; Larbi et al., 2022).

Integration of informal waste actors into municipal or centrally managed systems is one strategy to improve SWM; however there remain doubts if this is the most effective and ethical way to improve the livelihoods of informal waste actors, improve environmental services and produce economic benefits (Millington and Lawhon, 2019; Nandy et al., 2015). Sustainable entrepreneurship is often cited to deliver social, environmental and economic benefits (Sarkar and Pansera, 2017) and can be used to address societal challenges, such as plastic waste, through the development of new business models (Anabaraonye et al., 2022; Dijkstra et al., 2021). Sustainable entrepreneurship and informal waste management are two sides of the same coin, since the waste value chain contains many different formal and informal ventures seeking to process plastic waste and make money (Amankwaa and Oteng-Ababio, 2014; Oyake-Ombis et al., 2015). There are many examples in literature and practice of entrepreneurs providing needed waste management services while also turning out a profit (Gregori et al., 2019; Vital Ocean et al., 2020; WASTE, 2022). For example, a study into the SME Mr. Green Africa in Kenya demonstrated quality recyclate, business profits and social benefits can be achieved by plastic collection and processing ventures (Gall et al., 2020). However, entrepreneurs in the developing world also face challenges due to lack of resources, capacity, finance and knowledge on how to build successful businesses (Gabriel, 2016; Ratten, 2014).

This study develops a tool that supports bottom-up entrepreneurship solutions for plastic waste in LMICs, called the Plastic Venture Builder (PVB). This tool is designed to address several gaps in research and practice. First, we could not find a tool that was suitable for the complex and heterogeneous context of waste management in LMICs, meeting the call for ‘standardized and easy-to-apply low-cost methodologies . . . in low-and middle-income countries . . . encompassing multiple sustainability dimensions’ (Zurbrügg et al., 2014: 561). Thus, the tool uses success cases from a variety of geographies, company sizes and operating at different levels of the plastic value chain to capture the breadth of opportunity in this sector.

Second, a tool should be simple enough to be used by entrepreneurs, but comprehensive enough to provide actionable insights. Scientists have raised the importance of conducting ‘boundary-spanning’ research that is embedded within the empirical context and can provide practical advice, especially in the context of grand societal challenges (Bednarek et al., 2018). Especially since entrepreneurs in the developing world may be fragmented and not well connected to the support system, the study results can help new ventures access learnings and feedback and avoid reinventing the wheel.

Finally, the study adopts an entrepreneurial perspective, which is missing from studies that focus on technical or governance aspects of SWM. Support tools that are designed for municipalities or governments are ill-suited to aid entrepreneurs and there is a need for a framework that accounts for unique entrepreneurial challenges such as market conditions and attracting financing. Therefore, this paper focuses on identifying characteristics of successful plastic waste ventures in LMICs and operationalizing this into a tool for sustainable entrepreneurs. In the next section, we describe the methodology of the study. We then apply the PVB to three case studies in Africa to demonstrate the application of the tool. We further discuss implications of the methodology, applications of the tool and offer conclusions.

## Methodology

**Figure 1.**
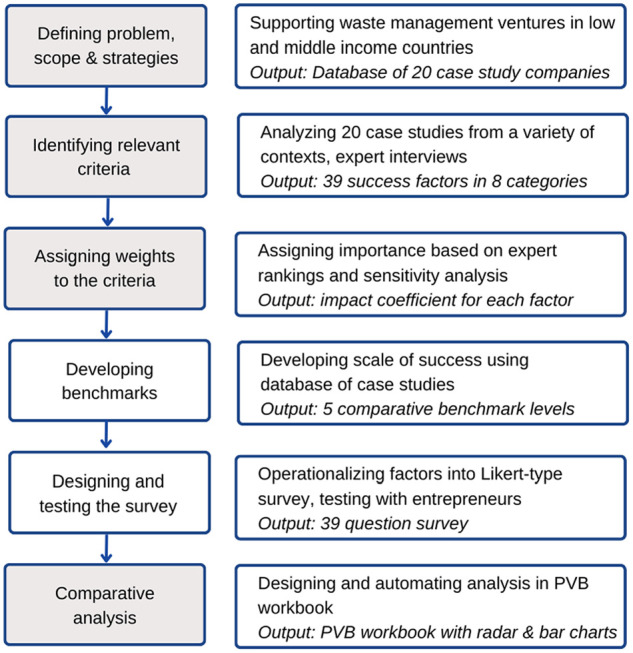
Methodological strategy for development of the PVB tool. The boxes shaded in grey are adopted from the MCDA methodology.

Developing the PVB tool was a multi-step iterative process that began with reviewing available tools and frameworks (Supplemental Materials). These frameworks were deemed inadequate because they were geared toward top-down decision makers or required data that may be inaccessible to entrepreneurs. Thus, we decided to create a rapid assessment tool that can be conducted by the entrepreneurs themselves, with immediate results and cover elements that are relevant for a business. We use the multi-criteria decision analysis (MCDA) as the framework for tool development. MCDA is a method used in environmental decision making to systematically analyze potential strategies along a set of pre-defined criteria (Achillas et al., 2013; Goulart Coelho et al., 2017; Khan and Kabir, 2020). MCDA steps include (1) identifying a problem, defining scope and potential strategies, (2) identifying relevant criteria for the problem context, (3) assigning weights related to the importance of each criteria, (4) scoring the strategies based on the criteria, (5) calculating a final ranking and (6) conducting sensitivity analysis and communicating results (Marazzi et al., 2020). We used the weighted sum model in our calculations, which involves multiplying the scores by the weights to get an overall score. The methodological steps of PVB development are shown in Figure 1, the grey boxes are steps taken from the MCDA.

The problem scope is the challenge of creating a successful plastic waste management venture in LMICs, with success being defined as a viable company delivering needed waste management services. Thus, success can look different in different contexts depending on the economic conditions and which waste services are demanded. We created a database of 20 small and diverse entrepreneurial ventures along the plastics management value chain in Asia and Africa (Supplemental Materials). Data was collected through semi-structure interviews and content analysis of documents related to the companies. We compiled a list of factors that were mentioned in the interviews or documents as contributing to the company’s success. Concurrently, academic and grey literature was consulted to find frameworks to organize our growing list of success factors (Guerrero et al., 2013; Zurbrügg et al., 2014). This process was a funneling of a wide variety of data and information toward a structured list of factors. The process involved combining similar factors, removing factors that were uncommon and separating factors that were overlapping. We then formally invited experts to offer feedback through online interviews to determine if the methodology was sound, and the list of factors was exhaustive based on their experience working with waste ventures in LMICs (see Supplemental Materials for expert details).

In discussions with experts, it was clear that some factors are more critical than others and should have a larger impact on the final assessment, which was facilitated using impact coefficients, also known as weights. To assign weights for each of the 39 factors, we qualitatively coded each category and factor as low, medium, or high importance based on expert input. We used these rankings to vary the weights per criteria. The final weights are shown in Table 1 as a percentage. We conducted a sensitivity analysis by varying weights, and the ranking of project scores was found to be robust (Supplemental Materials).

**Table 1.** Factors contributing to a successful plastic waste venture including survey questions and final weights.

Category	Factor	Operationalization	Weights in %
Politics	(Inter)national commitment	Is there a strong (inter)national commitment to plastic management?	1.00
	Local commitment	Is there a local governance commitment to solving the problem? Is this reflected by enforcement of policies? E.g. support of local mayors	1.00
	Prohibitive institutional context	Is the project free or not affected by corruption, lobbies, mafia influence? Is the project free from prohibitive conditions, for example an election year?	3.00
	Support from public sector (land, collection, etc., except for funding)	Is the government/municipality supporting the project?	2.00
	Government waste programme	The government/municipality has no competitive waste management scheme (at best it is complementary)?	3.00
	*Sum of weight for category*		*10.00*
Economics and Market	Market traction for final products	Is there a market? Are there (sufficient) buyers for your product(s) made of recycled plastics?	2.12
	Supply	Is there sufficient access to feedstock at a reasonable cost, no competition, etc.?	1.41
	Competition and alternatives	Is the product competitive compared to alternatives? Considering price, quality, ability to deliver, etc.	1.41
	Potential for scale-up	The project has growth perspectives (volumes, sales, geographic expansion)?	2.12
	Side economic activity	Is there a side activity (franchising, consulting, etc.) bringing in additional revenue?	1.41
	Market stability	Does the business have a stable portfolio of clients and steady income?	1.41
	Sufficient incentives along the value chain	Is each level of the value chain incentivized to participate / function effectively?	2.12
	*Sum of weight for category*		*12.00*
Financial sources	Access to plastic credit funding	Is the project leveraging plastic offset to help finance the operations?	1.57
	Corporate support (funding)	Does the project benefit from corporate sponsorships and funding?	1.57
	Subsidies from public sector	Is the government/municipality directly subsidizing the activity?	1.57
	Grants	Does the project benefit from any grant from individuals, incubators, international alliance or foreign government? (e.g. crowdfunding, startup prize, call for proposal, etc.)	1.57
	Loans	Are there loans available from (development) banks or international alliances?	1.57
	Financial ethics	Does the project ethically consider the source of funding? E.g. funding from chemical/oil and gas companies, pure greenwashing	0.79
	Self-funding	Can the project self-finance with limited external funding? E.g. Low capex with support for initial costs	2.36
	*Sum of weight for category*		*11.00*
Social	Communication/awareness raising	Does the project have a strong capacity to communicate/raise awareness while operating?	1.80
	Improved treatment of workers and waste pickers	Does the project improve treatment of workers or waste pickers, which could be in terms of wages, social protection, status, cooperatives, health, education, etc.	1.80
	Added value on current waste management	Does the project add waste management services or functions that are needed in the community?	1.80
	Involvement of the community in the project	Project designed for and with the local community? Considering the local religion, working culture, etc.	2.70
	Plastic pollution awareness	Is the population aware of the problem? Adjusting their behavior?	0.90
	*Sum of weight for category*		*9.00*
Technology	Fit-for-purpose technology or innovation	Is the project introducing a technology or innovation that increases effectiveness?	7.00
	Machines built on site	Are the recycling machines built at the local/national level instead of being imported?	2.33
	Product innovation or design	Is there a unique innovation regarding the final products, considering the local and regional context?	4.67
	*Sum of weight for category*		*14.00*
Operations	Local technical capacity	Does the project have the capacity to maintain and adapt recycling machines or equipment for its own purposes with limited resources? Are there in-house engineers or local technical partners?	2.60
	Transport infrastructure	Are there existing functioning transport systems that connect operations? Such as roads, shipping routes, etc. access to gasoline	1.73
	Access to electricity and water	Is there easy access to reliable energy/electricity and current water?	1.73
	Health or safety risks	Are the operations safe and well-managed in terms of health risks?	1.73
	Access to fit-for-purpose feedstock	Is there a reliable and stable access to fit-for-purpose feedstock for the operations?	2.60
	Ability to scale the operations	Does the project have the capacity to grow/scale the equipment and processing? (It can be internal or external capacity)	2.60
	*Sum of weight for category*		*13.00*
Team	Strong management team	Does the management team have what it takes to successfully lead the project? Is there a good balance between local expertise and experience and global perspectives and networks? Is there full local ownership to ensure continuity?	4.80
	Organizational capability	Has the team been properly chosen and trained for the job? Is there employee retention?	3.20
	Organizational credibility	Is the organization respected within the local context?	3.20
	Fruitful partnerships	Does the project have fruitful partnerships with other stakeholders in the ecosystem? E.g. business partners, local or international NGOs	4.80
	*Sum of weight for category*		*16.00*
Legal	Legally conscious	Is the project set up within the national and local regulations and legal requirements? The project has no legal risks?	9.00
	Favourable institutional context	Is there no potential for bureaucratic or legal obstacles to block the project?	6.00
	*Sum of weight for category*		*15.00*
	Total weight		100

NGOs: Non-governmental organizations.

The MCDA methodology is applied to decision contexts where multiple strategies or alternative pathways are compared to one another (Dean, 2022; Goulart Coelho et al., 2017). Since our tool is designed for entrepreneurs to understand their own strengths and weaknesses, in essence a single case study, we needed to integrate comparative benchmarks into the tool. This deviates from the traditional MCDA steps and we built our own benchmark model to compare companies with hypothetical reference points. We classified benchmarks as ideal, very good, good and insufficient (Supplemental Materials). We scored new test cases with the tool to check for external validity and found that successful cases were correctly identified by the PVB and properly ranked compared to benchmarks.

The PVB is intended to be used by entrepreneurs as a self-assessment methodology, and therefore we needed to operationalize our findings into a tool. We then converted each of the success factors into a simple and unambiguous question with response options on a five-point Likert scale from strongly agree to strongly disagree. The survey was tested in English and French with eight projects. Respondents were asked to judge their own company using the survey and provide feedback on the questions, method and interpretation of the results. During this process we slightly modified the phrasing of questions to improve comprehension. The final version of the survey contains 39 success factors clustered in eight categories and is shown in Table 1.

**Figure 2.**
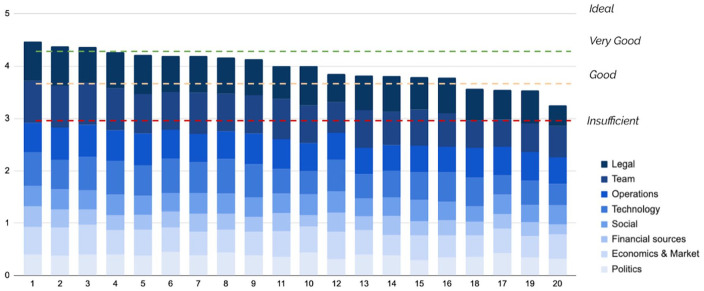
Overview of the 20 case study companies and their scores on the PVB broken down by category. The benchmark cutoff levels are shown and described in the Supplemental Materials.

After filling in the survey of the 39 questions, the PVB workbook automatically calculates the final score using the weighted sum model and compares the results to the benchmarks. The results are shown both as bar charts and as radar diagrams, which highlight how the venture scores on a category level. The PVB survey and workbook can be used for a single case study, filled in multiple times to compare projects or used to monitor progress over time. Figure 2 shows the PVB score results for the 20 case studies in the database, alongside the benchmark cutoffs.

## Case study application

### Case study context: sub-Saharan Africa

Here we present the results of three specific projects in order to concretely illustrate the output of the PVB and to demonstrate the key takeaways and points for future research. We use comparable projects of sub-Saharan ventures transforming plastic waste into paving blocks. In Africa, plastic consumption is much lower than other Western or developed nations, but this is projected to increase significantly in the coming years (Ayeleru et al., 2020; Babayemi et al., 2019; Sadan and De Kock, 2021). Plastic policies, such as bans and taxes, have been implemented across the continent with varying degrees of success, but there remains a need for projects to tackle an ongoing mismanaged waste problem (Chukwuone et al., 2022; Dumbili and Henderson, 2020). Studies have found the informal waste sector to be a large source of jobs and income and plastic management (Barford and Ahmad, 2021; Bening et al., 2021; Kaza et al., 2018). Scholars in Nigeria have called attention to the opportunity for entrepreneurs to work on the plastics value chain, noting the potential economic, social and environmental benefits, but recognize that more political support is needed (Anabaraonye et al., 2022). Viable business models focused on managing plastic waste in Uganda (Gregori et al., 2019), Kenya (Oyake-Ombis et al., 2015), Ghana (Obeng-Odoom, 2014), Mali and Senegal (WASTE, 2022) and many more exist in practice. Furthermore, international organizations have spearheaded entrepreneurial programmes aimed at plastic in the continent. There is momentum building for plastic waste ventures in Africa; however, there remain challenges for entrepreneurs due to lack of internal capacity and trainings, as well as external conditions, technological limitations and lack of funding.

The next section presents three case studies and applies the PVB to better understand the opportunities and challenges related to developing a waste management venture in LMICs. To apply the PVB, an entrepreneur or project manager fills in the 39-question survey as accurately as possible about the internal and external conditions of the venture. This gives the raw score, which is multiplied by the weight in order to get an overall weighted score. The PVB workbook automatically calculates the results and presents this alongside the benchmarks to give the respondent an idea of how their project fares in total and on a category level. These results are presented with raw numbers, but also bar charts and radar diagrams to easily visualize strengths and weaknesses. The results can be used to strategize improvements to the business model, monitor the company over time, or compare multiple projects. For the three case studies introduced, the entrepreneur filled in the PVB survey themselves, and the analysis was discussed with the researchers.

### Company 1: Project Ivory Coast

This company has been operating in Ivory Coast since 2015 and when the initial plastics-to-flakes activity reached a profit, the company began developing other products including paving blocks for residential areas and sidewalks. Though the company was initially able to diversify and expand operations, the company is now struggling with production. This is due to limited production capacity as a result of locally manufactured machines that are not properly suited for the processes, as well as inadequate maintenance. The company has been self-funded, and the team leader is highly resilient and passionate. Market traction is developing and there is potential to overcome their production obstacles, but this will be challenging. We assessed the company using the PVB model and Figure 4 shows one of the PVB outputs, a radar diagram of the results relative to the benchmark projects. The radar diagram uses a percentage scale with 100% being the highest score possible and 0 being the lowest. The case study score is highlighted in thick black line, and the diagram shows how the venture scores compared to the hypothetical benchmarks of ideal, very good, good and insufficient.

**Figure 3.**
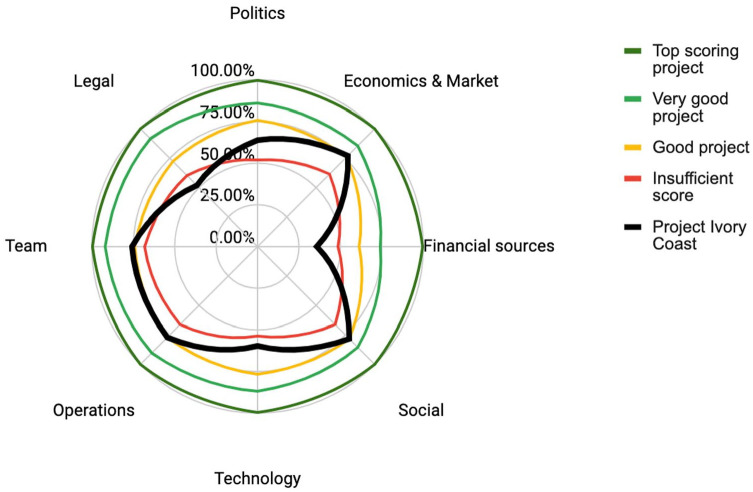
Radar diagram of Company 1: Project Ivory Coast.

As shown in Figure 3, a main bottleneck for the Company 1 is lack of financial sources and legal challenges, which score poorly and below the sufficiency benchmark. At the time of the interview, the company was aware of their challenges and was reaching out to international players looking for financial options. The local recycling experience over the past 7 years has also strengthened the company’s reputation and its capacity to find clients for paving blocks, but other sources of financing are needed. Considering the poorly scored legal category, interviews confirmed that the company is currently deemed a ‘cooperation’ and not a proper registered private company (e.g. Limited Liability Company). Because of this, the land used for their operations is granted by the government but can be taken back at any moment or other rights can be claimed on their operations. Interestingly, the low legal score was also related to access to funding. The project was in discussions to access corporate social responsibility (CSR) financial support; however, the corporate partner abandoned the project after due diligence revealed the informal legal structure. The PVB results confirms that legal conditions can be a major barrier for a company’s continued success.

The technology has been adequate thus far, but the assessment suggests there remains room for significant improvement. Interviews reinforce that the company has slow organic growth, which can be attributed to a huge lack of engagement from the public sector and an unsupportive political environment. The company has been operating with limited top-down support, making it hard to accelerate the company’s expansion. Shifting attention to focus on synergies with major corporations as clients and accessing funding to scale-up production could strongly help unlock this company’s potential. The company appears to be operating sufficiently in terms of team, operations and social considerations.

### Company 2: Project Guinea

**Figure 4.**
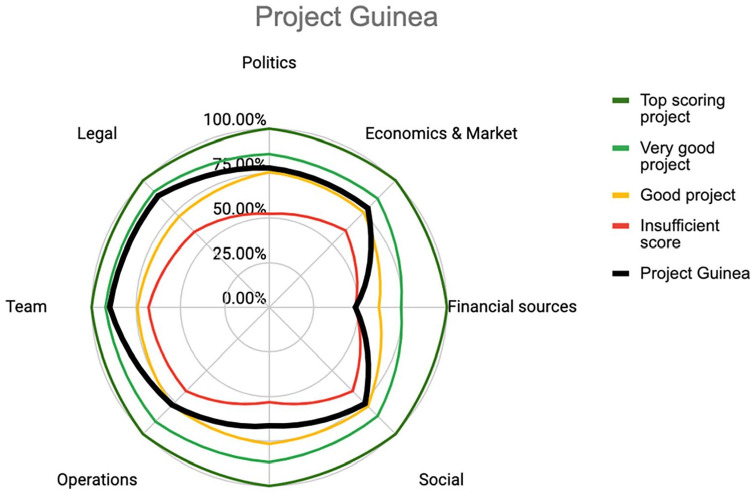
Radar diagram of Company 2: Project Guinea.

This second company is operating in the Republic of Guinea and is focusing on paving blocks for the construction sector. Founded by a passionate woman in 2019, the project gained traction and visibility through startup competitions, quickly building a favourable ecosystem of partners. With limited resources and seed funding, the team managed to complete a proof-of-concept and validate first buyers. With very basic and low-tech equipment, the production facilities are currently able to produce 200 pavers per day and this includes an occasional night shift which was added to increase production. In the current context, the machines and processing seem to have reached their limit. The team is at a point where they must decide if and how to scale up the production to meet the high potential of the demand from construction companies. Additionally, the pavers are competitive with the alternatives made of concrete and it is unknown the exact size of the potential market. The team has grown to nine people to date, mainly young local women. In Figure 4, the radar diagram the radar diagram shows the results of the PVB for Company 2 compared to the benchmarks.

The assessment is relatively positive, though some areas present a risk. The company scores poorly in financial sources and access to capital, and this could lead to a bottleneck in the company development. Interviews suggest that the public sector and other financial institutions are reluctant to provide subsidies or funding because this is a private-led business and not a public programme. Other types of financing are needed to industrialize production, but the company has minimal experience, although recent collaborations with international organizations may lead to funding to upgrade the recycling line. Technology-wise, the team benefits from more advanced engineering skills, but has no in-house manufacturing capabilities, for which they rely on external help. These two low-scoring categories suggest that building out technical expertise and securing investments are needed to move forward.

On a positive note, the team is well-connected and has mentorship providing economic, financial and strategy advice. Operationally, the team is experienced with feedstock procurement, team management and has the tools to handle logistics. Given current production capacity and local demand, the economics are sound, though there is potential to expand pending investments. The local political and legal environment is positive and benefits from strong connections with relevant ministries and institutions. The company is registered properly, though future expansions may require new licenses.

### Company 3: Project Kenya

**Figure 5.**
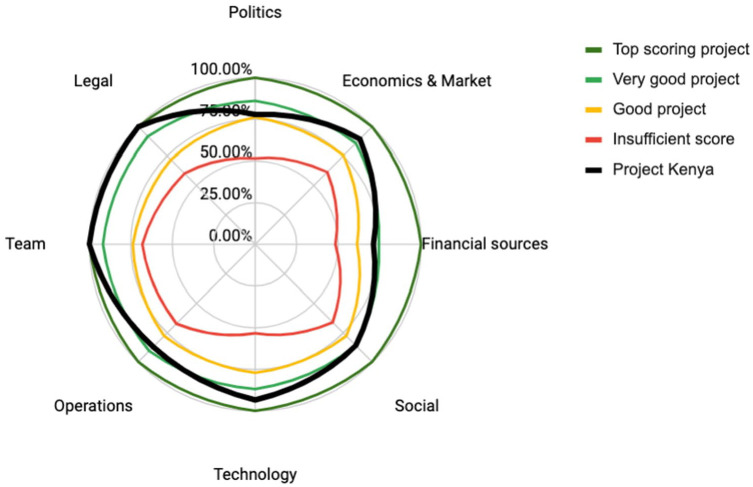
Radar diagram of case study 3.

The third company is operating in Kenya and started in 2017 to produce paving blocks and bricks for the construction and housing industry. Led by a very dedicated woman, the project received local and then international visibility, attracting new stakeholders and launching new opportunities for scale-up. Today, the company is still developing locally and organically, producing more than 2000 pavers per day with consistent upgrading of equipment and strong market traction. In addition, expansion to other sub-Saharan countries is pending; Figure 5 shows the radar diagram for Company 3 produced by the PVB.

What is immediately apparent is high scores across the categories, with politics receiving a lower, but still sufficient, grade. In Kenya, there is an increasingly favourable environment for waste management with a relatively high maturity in the sector among sub-Saharan countries. Internally, the team leader is a huge asset, she has a strong vision with a ‘doer’ mindset paired with sufficient technical skills and resilience for the challenges of entrepreneurship. The company has been able to access financing from multiple sources, which has expanded after a successful proof-of-concept demonstrated the business viability. However, it is recognized that in order to expand to new markets, even more financing is needed. Currently, most growth is organic, and more aggressive upscaling will require larger investments. Similarly, the current market has been studied extensively which has led to the success of marketing pavers to the housing and construction market. Expansion will thus require analysis to understand if the model can be replicated in new markets or if adjustments for new ecosystems are necessary.

Presently, the company has benefited from strong in-house engineering capacity allowing the team to build and maintain machines, adjust them to changing needs and scale up to meet increasing demand. Thus far, there has been a focus on operations, technology and production, and there is room for improvement on the social side. However, the project has raised awareness, and inspired a generation of young entrepreneurs to follow suit.

### Cross-case comparison

Individually, insights generated from the assessment tool have shed light on specific shortcomings and strong points from the three companies relative to the benchmarks. There is also the added benefit of being able to compare case studies with each other, while considering each country has a unique political, cultural and financial environment. In Table 2, we present a cross-case comparison of these cases, including a colour coding to visualize how the scores compare to the hypothetical benchmark levels.

**Table 2.** Cross-case comparison per category for Sub-Saharan African case studies. The colours are aligned with the benchmark levels included in the PVB workbook.

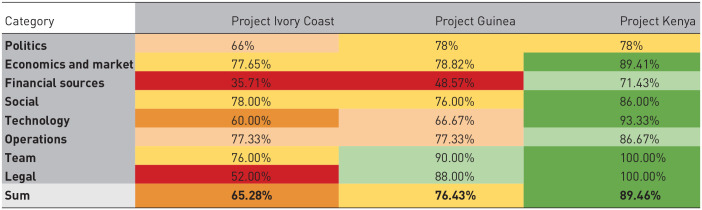
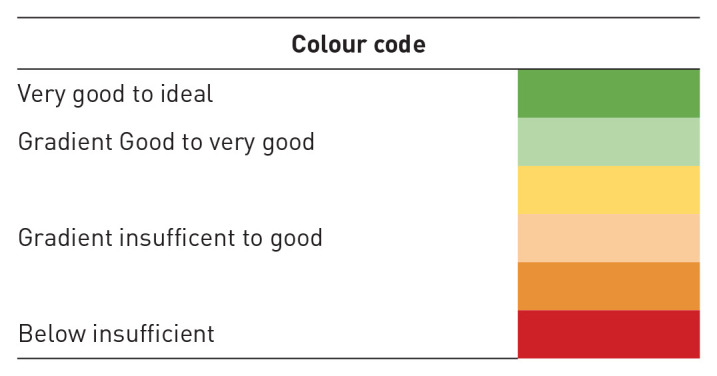

Evidently, the project in Kenya is the most and the Ivory Coast project the least successful. These companies have different external conditions, and the legal challenges in the Ivory Coast are related to the regulatory structure there, whereas in Kenya there appears to be no regulatory blocks. Some of these conditions, such as political environment, are hard for an entrepreneur to influence; however results can encourage ventures to invest in certain areas of business development. For example, conducting more market research and spending resources on building technological capacity would benefit Company 1 and 2 and has contributed to the success of Company 3. The cases score relatively poorly on the financial resources criteria, due to limited options in the African context. However, by going through the PVB survey and workbook, the process has the added benefit of prompting entrepreneurs to consider a wider range of financing than they are used to, such as plastic credits, CSR support, venture capital or loans. Especially for companies seeking to scale, innovative approaches to financing can help them meet expansion goals.

The comparison also demonstrates there is no one-size-fits-all approach to build a successful plastic waste venture. Company 1 has had a stronger focus on the social aspects of their company, and on a small scale has succeeded in promoting social benefits and introducing a new product to the local market, while struggling with less favourable political and legal conditions and technological limitations. Company 2 is focusing on building their team and operations despite limited finances and technological capacity and has demonstrated a market case for the company. And finally, Company 3 is farther along in business development and scoring positively, which also strengthens the case for expanding and seeking additional financial sources.

## Discussion

### Importance of success factors

After conducting the extended case studies and seeing patterns emerge, we decided to revisit the initial sample of 20 companies across Africa and Asia and determine which success factors were more important for overall success. If some conditions are more impactful than others, then entrepreneurs can prioritize resources and time on them to increase their chance of success.

We consider the team to be the most critical factor, which is a common conclusion about entrepreneurship in general. No matter how good a project is, if the team is not suitable, strong or dedicated enough to carry it forward, the chance of success drops accordingly (Tseng et al., 2021). Previous studies have called attention to the importance of knowledge, skills, team capacity and other ‘human factors’ in effective waste management (Sodhi et al., 2020; Zurbrügg et al., 2014).

Next, the categories of legal, technology, economics and market are the second level of important factors and high scores in these groups are good indicators of a project’s success. Considering the case studies, a strong and suitable technology can catalyze success quite fast, and scholars have noted that technological improvements, even marginal ones, can significantly increase waste management capacity (Millington and Lawhon, 2019). Similarly, with legal, if a company is well structured, resilient and aligned with regulatory requirements, this can be the difference between success and failure (Bufoni et al., 2016). Functioning well within the market and developing a revenue model are needed for the company to be profitable and viable (WASTE, 2022). Operations can be a bottleneck, as a company may function effectively on a small scale yet run into operational challenges with growth. Operational factors also involve external conditions and infrastructures that the venture may not be able to influence, but can support or limit its development (Guerrero et al., 2013).

Finally, social, political and financial sources are still important but their contribution to the venture’s success is not as definitive. Social factors do not seem to be linked to project success, however it is worth exploring if business success leads to increased social benefits (Gutberlet et al., 2017). Politics is rarely a strong asset or focus of new ventures, however this factor can turn out to be a major barrier if a competitive scheme or mafia-like organization runs waste management operations in the region. Conversely, studies have demonstrated that political shocks or disruptions may also serve as ‘windows of opportunity’ for entrepreneurs (Véron et al., 2018). Finally, financial sources scores appeared to be less important for young ventures. This does not mean that finance is not important, but rather that accessing diverse funding sources comes after building a strong core team, being legally on track with a defined solution and having a concrete business model. In essence, a business plan comes before an investor. Some analyzed cases did find financing to be a major obstacle, and this could be attributed to weaknesses in other categories, such as missing a capable team member to raise funds.

Though we have classified these factors into three groups to ease prioritization, there are interrelationships between the factors that could contribute to synergies or exacerbate challenges. For example, the one between political and legal factors; if there is a favourable political climate it is logical that legal issues would be minimized, and in an unfavourable political environment, legal challenges may arise. The Supplemental Materials includes the PVB analysis of the 20 case study ventures as well as Spearman’s correlation analysis between the factors and overall scores.

### Applications of the PVB and outlook

During the research and development process of the PVB, the tool was used by partner companies to evaluate their progress and consider pathways forward. For example, after being shown the results, the company in Ivory Coast realized the correlation between finance and legal structure, which was overlooked in the past and registered as a for-profit company. This led to sponsorships to buy new equipment, which in turn increased their visibility and social impact. The Guinean project leveraged their local reputation toward international donors, allowing them to scale-up their current recycling infrastructure to increase their production capacity. Additionally, they are working to pilot plastic credits as additional financing, which will incentivize waste pickers and secure more quality feedstock for scaling up. The PVB is a useful tool for engineering consultancy as well. Traditionally, expensive pre-feasibility studies are required by funders and institutions before implementing projects. Since the PVB maps all the main components of a project, it can be used to quickly conduct feasibility or screening studies on any number of case studies. Thus, the PVB can be a way to increase the success rate of ventures, reduce the costs and burdens of screening studies and can be used to monitor progress with a measurable score.

A spreadsheet version of the PVB tool is included in the Supplemental Materials of this article and will soon be hosted free online. As more entrepreneurs fill in the PVB, provide feedback and generate a larger sample of case studies, the PVB will continue to be improved to maximize its utility. Further, with more case studies, wider trends can be ascertained, such as differences based on geographies, value chain targeted or the age of the company. If specific regions or types of companies face similar challenges, policy makers and support agencies can direct their attention and resources accordingly. This will facilitate tailored support for social goals of supporting entrepreneurs and managing plastic waste. Thus, an important next step in this research is identifying support mechanisms that can be linked to each of the success factors and identify both what an entrepreneur can do to improve their results and what development and aid agencies can do to support ventures. Finally, the tool has been designed with plastic waste management in mind, but with a few modifications to the technology and operations factors, can be easily applied to social enterprise contexts besides waste.

## Conclusions

The PVB is a rapid assessment tool designed to be simple and widely applicable, while also providing meaningful results to entrepreneurs, project managers and decision makers. The framework was designed to be applicable across a range of heterogeneous contexts, therefore assumptions were essential, and the tool cannot reflect all the nuances of reality. Future research is needed to see if the tool accurately captures failed companies, as well as those in a pre-development stage. Furthermore, the factors identified in our study are interconnected and we could only approach this superficially in the tool development (Bufoni et al., 2016; Mwanza and Mbohwa, 2017). Another challenge is that entrepreneurs can self-assess their own projects, which can lead to issues if the assessor does not accurately score themselves. In a few of our test cases, we found entrepreneurs to be overly optimistic about their projects, scoring themselves higher than the researchers did. This shows that there will be inherent subjectivity in the results depending on who is filling in the PVB.

Our results demonstrate that success as a profitable plastic management company in LMICs can be reached multiple ways, and there is not one guaranteed path to create a viable waste management venture. We identified critical conditions in the categories of politics, market and economics, financial sources, social, technology, operations, team and legal. However, these factors can be prioritized, and results suggest any new venture begins by creating a strong team with a solid technology that meets legal conditions of the local area. Then, the focus can turn toward operations and understanding the economics and market. Interestingly, access to finance is one of the categories least correlated with success and seems to be a project enabler rather than the key ingredient, essentially: finance will not determine a project success, rather a successful project will attract finance.

## Supplemental Material

sj-docx-3-wmr-10.1177_0734242X231180648 – Supplemental material for Plastic Venture Builder (PVB): An empirically derived assessment tool to support plastic waste management ventures in low- and middle-income countries Supplemental material, sj-docx-3-wmr-10.1177_0734242X231180648 for Plastic Venture Builder (PVB): An empirically derived assessment tool to support plastic waste management ventures in low- and middle-income countries by J.B. Grassin and H. Dijkstra in Waste Management & Research

sj-xlsx-1-wmr-10.1177_0734242X231180648 – Supplemental material for Plastic Venture Builder (PVB): An empirically derived assessment tool to support plastic waste management ventures in low- and middle-income countries Supplemental material, sj-xlsx-1-wmr-10.1177_0734242X231180648 for Plastic Venture Builder (PVB): An empirically derived assessment tool to support plastic waste management ventures in low- and middle-income countries by J.B. Grassin and H. Dijkstra in Waste Management & Research

sj-xlsx-2-wmr-10.1177_0734242X231180648 – Supplemental material for Plastic Venture Builder (PVB): An empirically derived assessment tool to support plastic waste management ventures in low- and middle-income countries Supplemental material, sj-xlsx-2-wmr-10.1177_0734242X231180648 for Plastic Venture Builder (PVB): An empirically derived assessment tool to support plastic waste management ventures in low- and middle-income countries by J.B. Grassin and H. Dijkstra in Waste Management & Research
