# Supplementary material for: Plastic Venture Builder (PVB): An empirically derived assessment tool to support plastic waste management ventures in low- and middle-income countries
Source: Waste Manag Res. 2023 Jun 30;42(3):232–43. doi: 10.1177/0734242X231180648 (PMC10905985; doi:10.1177/0734242X231180648)
Supplement: sj-docx-3-wmr-10.1177_0734242X231180648 – Supplemental material for Plastic Venture Builder (PVB): An empirically derived assessment tool to support plastic waste management ventures in low- and middle-income countries [file sj-docx-3-wmr-10.1177_0734242X231180648.docx]

**Supplementary Materials**

**I – Review of waste management tools and frameworks**

| **Tool** | **Assessment scope** | **Description** | **Data and methods** | **Context** | **Source** |
| --- | --- | --- | --- | --- | --- |
| Plastic Drawdown | Policy instruments for plastic waste reduction | Rapid assessment approach to evaluate national plastic waste generation and pollution, and analyses the potential effectiveness of different policy instruments in reducing waste leakage into aquatic environments | - Plastic waste flows and stocks - Expert group consultations - Modeling of BAU and policy instruments | Maldives | (Royle et al., 2022) |
| Integrated Assessment Scheme (IAS) | Sustainable MSW scenarios | (IAS) developed to simultaneously evaluate the economic, environmental and social dimensions in order to support the decision-making process and promote sustainable waste management in LMICs | - Scenario development - Field visits and interviews - Stakeholder consultations   MCDA support analysis | Bosnia-Herzegovina and Mozambique | (Perteghella et al., 2020) |
| ISSOWAMA framework | SWM projects in development | A project-specific approach to assess typical success or failure factors covering social, institutional and economic elements | - 28 item questionnaires - Field visits and semi structured interviews - Comparison with other projects | Indonesia | (Zurbrügg et al., 2012) |
| The STAR model | Clean Development Mechanism (CDM) waste management projects | Conceptual model of 5 waste management barriers and 15 potential linkages | - Qualitative review of 432 registered CDM waste management projects | Global | (Bufoni et al., 2016) |
| Waste management system performance | SWM in developing cities | 6 factor model influencing SWM: technical, environmental, financial, socio-cultural, institutional and political | - Literature review - Field visits and 122 item questionnaire - Workshops and interviews | 36 cities | (Guerrero et al., 2013) |

**II – Database of ventures used for PVB development. *(Micro: 1-3 employees, small: 4-25 employees, medium: 25-100 employees)***

| **Number** | **Region** | **Year founded** | **Size** | **Description** |
| --- | --- | --- | --- | --- |
| 1 | Asia | 2018 | Medium | Transformation of most plastics and “non recyclables” into ecoboards, shaped and assembled into furniture |
| 2 | Asia | 2018 | Small | Transforming low value plastics and “non recyclables” into fuel via pyrolysis |
| 3 | Africa | 2018 | Small | Transforming LDPE, HDPE and PP into paving blocks (mixed with sand) |
| 4 | Africa | 2015 | Small | Turning plastic waste into flakes and paving blocks |
| 5 | Africa | 2018 | Micro | Collecting, sorting and selling plastics in bulk and developing lines to turn plastic waste into ecoboards and planks |
| 6 | Africa | 2018 | Medium | Turning plastic waste into bricks for housing |
| 7 | Africa | 2020 | Micro | Developing a solution to turn plastic waste for fishing industry into local products |
| 8 | Africa | 2020 | Small | Transforming plastic waste into paving blocks  and bricks |
| 9 | Asia | 2017 | Medium | Upcycling plastics in addition to wide waste management services |
| 10 | Asia | 2020 | Small | Collecting plastic waste from island and coastal communities, transforming it into flakes and pavers |
| 11 | Asia | 2011 | Small | Transforming plastic waste into a wide range of indoor and outdoor furniture (school chairs, benchess, etc) |
| 12 | Asia | 2018 | Small | Transforming plastic waste into flakes,  ecolumbers to build emergency housing, ecoboards |
| 13 | Middle-East | 2014 | Small | Upcycling all types of waste, especially plastics into ecoboards for vertical farming, outdoor furniture (tables etc) |
| 14 | Africa | 2021 | Micro | Developing solutions to turn plastics into bricks, bins |
| 15 | Africa | 2021 | Small | Managing a landfill, sorting plastics and baling and developing a system to upcycle plastics on site |
| 16 | Africa | 2020 | Small | Upcycling plastics into small products and furniture |
| 17 | Asia | 2016 | Small | Collecting waste in remote islands and transforming it into fuel and shipping valuable plastics |
| 18 | Africa | 2010 | Medium | Transforming plastic waste into iconic end-user products (big molded bins, basins, etc) |
| 19 | Africa | 2018 | Medium | Transforming plastic waste into high value flakes |
| 20 | Africa | 2020 | Micro | Upcycling plastic waste into small products |

**III – List of anonymized experts and their role in PVB development**

| **Expert role** | **Methodological brainstorm** | **Input on criteria and their relative importance** | **Feedback on final tool** |
| --- | --- | --- | --- |
| Expert on sustainable plastic solutions | x | x |  |
| Waste management and development expert |  | x |  |
| Small island developing state (SIDS) waste management specialist |  | x |  |
| Chief Technology Officer for a company developing low-tech / semi-industrial plastic recycling machines | x | x |  |
| Founder of waste management company in South East Asia |  | x |  |
| Professor of solid waste management with a focus on developing countries | x |  | x |
| Social innovation expert within a philanthropy organization |  |  | x |
| International solid waste expert and plastic recycling adviser |  |  | x |

Overall, the 39 criteria were added, defined and refined at first by the authors from the study of the main sample of projects, then challenged by the experts and complementary projects. Some criteria were deleted, others were merged, and some added and modified to come up with an exhaustive and balanced list of criteria. It was the same process for the categories of criteria themselves. We kept a detailed record of the changes, and this is available upon request.

**IV – Benchmark development and cross case comparison**

Using the diverse sample of companies described in Appendix II, we used the scoring method to give a value from 0 to 5 for each criterion for each project. This was done to check how companies scored on the factors and to define benchmarks based on more and less successful companies. We found it was unlikely for companies to score ‘5’s and thus created empirical benchmarks called “very good”, “good” and “insufficient”. These were defined using personal experience with the database companies from interviews ad document review, and knowledge about their effectiveness and challenges. These were adjusted by iteration with the goal to make the scales as comprehensive and close to reality as possible. The details of the calculation and specific values per benchmark can be found in the Supplementary Materials workbook.

The goal of the benchmarks is so projects can be judged independently from others. The overall benchmark lines are shown below, however there are different benchmarks for each group of criteria. As such, a project be deemed as ‘good’ overall, but may still have some categories score below ‘insufficient’ and vice versa.


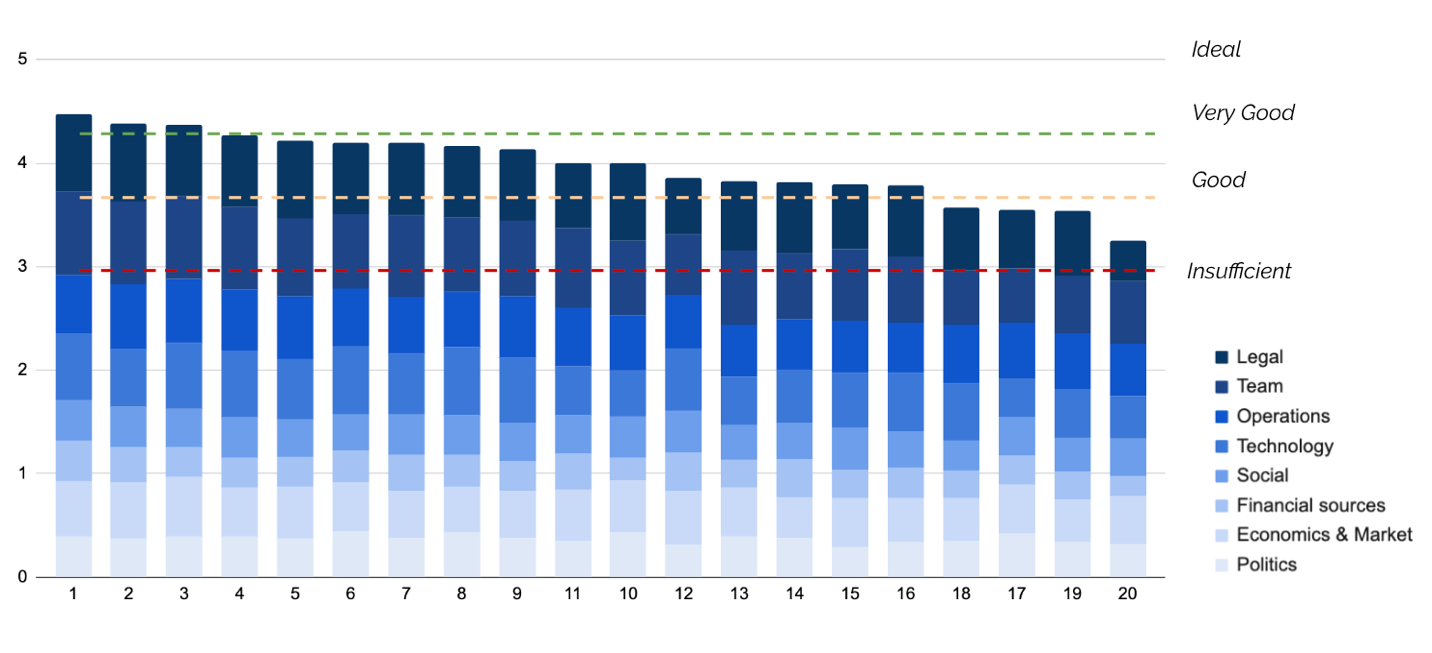


To visualize in a different way, a heat map shows the projects ranked 1 to 20 (based on their overall score) and shows the breakdown of their strengths (green) and weaknesses (red) with a gradient of color. This is also a way to visualize criteria most correlated with success and demonstrates how some high scores can compensate for lower scores.


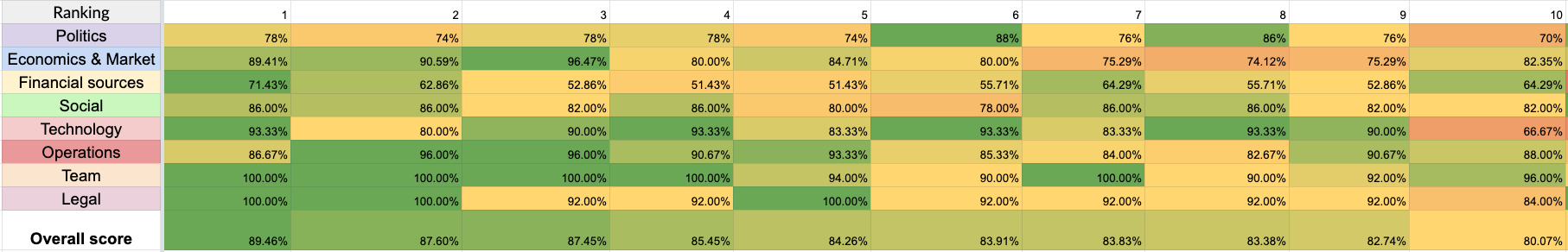

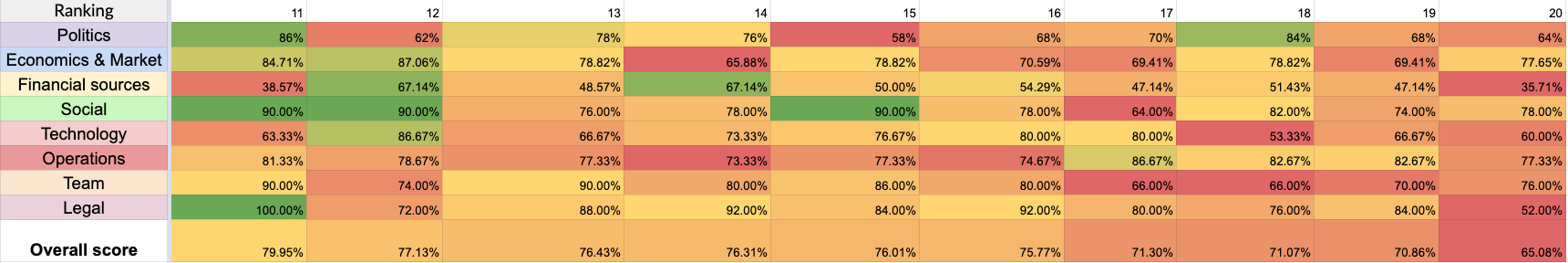


**V – Sensitivity analysis**

Regarding the weights of the eight categories and 39 drivers, we also used an iterative process. We started by allocating the same weight to each driver and viewing the results, which was not an accurate representation of the spread of case study companies. Thus, we used expert feedback to make a differentiation. Depending on how experts judged each driver, we gave a weight of 1, 2 or 3 (low, medium or high importance) to reflect how influential for the success of a project the driver is (either positively and/or negatively). For instance, the factor “prohibitive institutional context” has a weight of 3 because it affects greatly the outcome of the project. Comparatively, the (inter-)national commitment has only a weight of 1 because it is a “good to have” but not a “must have” as many projects succeed without it. After we had ranked the drivers, we did a similar exercise for the factor categories. This gave us a wide spread of weights. We then used the Spearman’s coefficient analysis to judge which categories had most influence on the final rankings and to validate that our weights were robust.

Within the MCDA methodology, one critical aspect is the weights given to each criteria. Changes in the weights have the potential to influence the outcomes and overall rankings of results. We ran a sensitivity analysis on the 20 case studies to see if our weights were robust or if slight changes in the weights led to large fluctuations in the final scores. We created hypothetical alternative weighting scenarios, with an emphasis on different categories such as a focus on the financial categories (economics/ market and financial sources), on technical categories (technology and operations) and on people related categories (team, social and politics).

| **Sensitivity run** | **Politics** | **Econ. & Market** | **Financial sources** | **Social** | **Tech** | **Ops** | **Team** | **Legal** | **Spearman’s Rho** |
| --- | --- | --- | --- | --- | --- | --- | --- | --- | --- |
| Emphasis Economics & Finance | 8 | **16** | **16** | 10 | 13 | 12 | 14 | 11 | 0.988 |
| Emphasis Tech & Operations | 8 | 11 | 10 | 9 | **16** | **16** | 14 | 11 | 0.992 |
| Emphasis Team, Social, Politics | **13** | 11 | 10 | **17** | 11 | 12 | **17** | 11 | 0.980 |
| Final weights | **10** | **12** | **11** | **9** | **14** | **13** | **16** | **15** | **1** |

The modified weights had only a slight impact on the final scoring and rankings of the projects. This suggests the PVB is a robust tool that is not overly sensitive to changes in impacts per category. The correlation coefficient demonstrates that the rankings are over 98% compatible with each other.

**VI – Analysis of correlation – Spearman’s Rho**

| **Variables** | **Values** | **Politics** | **Econ. & Market** | **Financial sources** | **Social** | **Tech** | **Ops** | **Team** | **Legal** | **Total score** |
| --- | --- | --- | --- | --- | --- | --- | --- | --- | --- | --- |
| **Politics** | Spearman's rho | 1 | 0.187 | 0.092 | 0.095 | 0.3 | 0.235 | 0.337 | 0.479** | 0.456** |
|  | Sig. | . | 0.429 | 0.701 | 0.69 | 0.199 | 0.318 | 0.147 | 0.033 | 0.043 |
| **Economics & Market** | Spearman's rho | 0.187 | 1 | 0.218 | .536** | 0.234 | 0.523** | 0.569*** | 0.351 | .664*** |
|  | Sig. | 0.429 | . | 0.355 | 0.015 | 0.321 | 0.018 | 0.009 | 0.129 | 0.001 |
| **Financial sources** | Spearman's rho | 0.092 | 0.218 | 1 | 0.32 | 0.497** | 0.137 | 0.399* | 0.293 | 0.524** |
|  | Sig. | 0.701 | 0.355 | . | 0.17 | 0.026 | 0.565 | 0.082 | 0.21 | 0.018 |
| **Social** | Spearman's rho | 0.095 | 0.536** | 0.32 | 1 | 0.262 | 0.127 | 0.413* | 0.274 | 0.445** |
|  | Sig. | 0.69 | 0.015 | 0.17 | . | 0.265 | 0.595 | 0.071 | 0.242 | 0.049 |
| **Technology** | Spearman's rho | 0.3 | 0.234 | 0.497** | 0.262 | 1 | 0.472** | 0.523** | 0.441* | 0.728*** |
|  | Sig. | 0.199 | 0.321 | 0.026 | 0.265 | . | 0.036 | 0.018 | 0.052 | 0 |
| **Operations** | Spearman's rho | 0.235 | 0.523** | 0.137 | 0.127 | 0.472** | 1 | .640*** | 0.406* | .708*** |
|  | Sig. | 0.318 | 0.018 | 0.565 | 0.595 | 0.036 | . | 0.002 | 0.076 | 0 |
| **Team** | Spearman's rho | 0.337 | 0.569*** | 0.399* | 0.413* | 0.523** | 0.640*** | 1 | 0.696*** | 0.899*** |
|  | Sig. | 0.147 | 0.009 | 0.082 | 0.071 | 0.018 | 0.002 | . | 0.001 | 0 |
| **Legal** | Spearman's rho | 0.479** | 0.351 | 0.293 | 0.274 | 0.441* | 0.406* | 0.696*** | 1 | 0.753*** |
|  | Sig. | 0.033 | 0.129 | 0.21 | 0.242 | 0.052 | 0.076 | 0.001 | . | 0 |
| **Total score** | Spearman's rho | 0.456** | 0.664*** | 0.524** | 0.445** | 0.728*** | 0.708*** | 0.899*** | 0.753*** | 1 |
|  | Sig. | 0.043 | 0.001 | 0.018 | 0.049 | 0 | 0 | 0 | 0 | . |

* Correlation is significant at the 0.05 level (2-tailed).

** Correlation is significant at the 0.05 level (2-tailed).

*** Correlation is significant at the 0.01 level (2-tailed).

**VII – Visualization of the results – Radar Diagram**

After several tests, the radar diagram has been chosen as the best way to visualize the main results from the tool. With a quick look, it is possible to understand in which categories a project has strengths and weaknesses and compare in each category to the benchmark. This diagram can be generated in Excel or Google Sheets with the option radar or spider diagram.

**
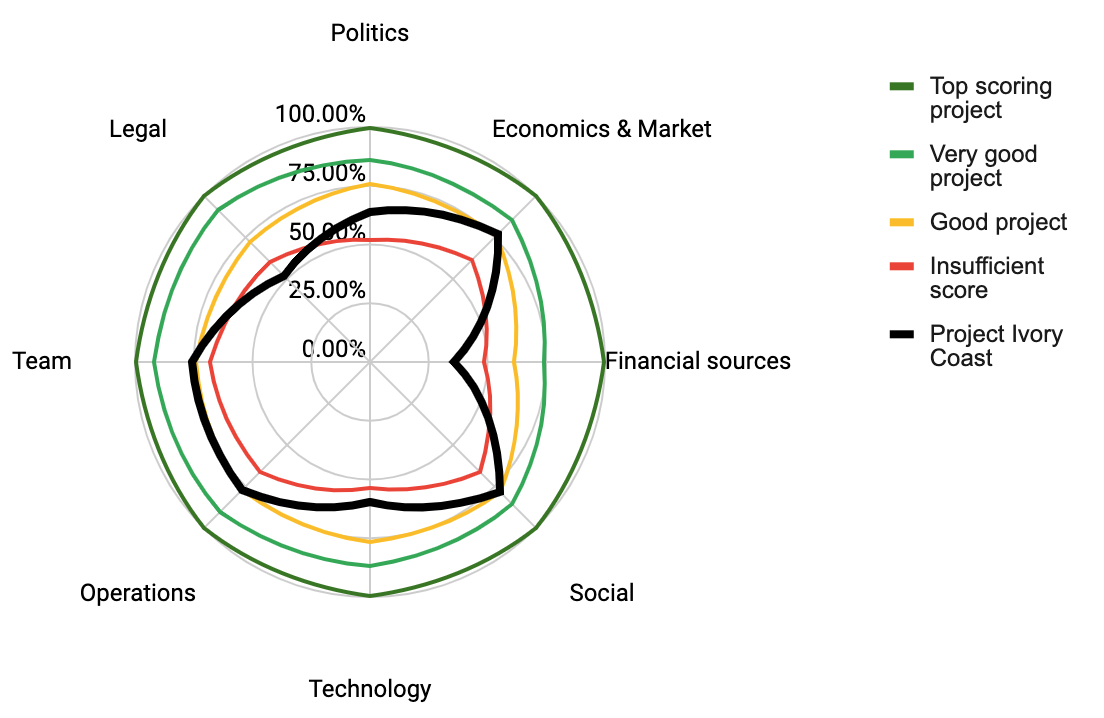
**
